# Continuous theta burst stimulation-induced suppression of the right fronto-thalamic-cerebellar circuit accompanies improvement in language performance in poststroke aphasia: A resting-state fMRI study

**DOI:** 10.3389/fnagi.2022.1079023

**Published:** 2023-01-12

**Authors:** Kai Zheng, Xinlei Xu, Yingying Ji, Hui Fang, Fanglan Gao, Guilan Huang, Bin Su, Li Bian, Guofu Zhang, Caili Ren

**Affiliations:** ^1^Department of Neurorehabilitation, Wuxi Central Rehabilitation Hospital, The Affiliated Mental Health Center of Jiangnan University, Wuxi, Jiangsu, China; ^2^The Affiliated Mental Health Center of Jiangnan University, Wuxi, Jiangsu, China

**Keywords:** aphasia, language, continuous theta burst stimulation, resting-state fMRI, randomized controlled trial

## Abstract

**Background:**

Continuous theta burst stimulation (cTBS) is a specific paradigm of repetitive transcranial magnetic stimulation (rTMS) with an inhibitory effect on cortical excitability for up to 60 min after less than 1 min of stimulation. The right posterior superior temporal gyrus (pSTG), homotopic to Wernicke’s area in the left hemisphere, may be a potential stimulation target based on its critical role in semantic processing. The objective of this study was to explore whether cTBS over the right pSTG can promote language improvements in aphasic patients and the underlying mechanism.

**Methods:**

A total of 34 subjects with aphasia were randomly assigned to undergo 15 sessions of either 40-s inhibitory cTBS over the right pSTG (the cTBS group) or sham stimulation (the sham group), followed by 30 min of speech and language therapy. Subjects underwent resting-state functional magnetic resonance imaging (rs-fMRI), and the aphasia quotient (AQ) of the Chinese version of the Western Aphasia Battery (WAB) was calculated before and after the intervention. This randomized controlled trial was registered in the Chinese Clinical Trial Registry (No. ChiCTR210052962).

**Results:**

After treatment, the language performance of the cTBS group was higher than that of the sham group in terms of the WAB-AQ score (*p* = 0.010) and the WAB scores for auditory comprehension (*p* = 0.022) and repetition (*p* = 0.035). The fractional amplitude of low-frequency fluctuations (fALFF) was significantly decreased in the pars triangularis of the inferior frontal gyrus (IFG), right middle frontal gyrus, right thalamus, and left cerebellar crus I. Clusters in the left orbitofrontal cortex exhibited increased fALFF. The change in WAB comprehension scores were significantly correlated with the change in the fALFF of the right IFG pars triangularis in both groups. Greatly increased functional connectivity was observed between the right pars triangularis and left paracingulate gyrus and between the right pSTG and right angular gyrus and the posterior cingulate gyrus with pre-and post-treatment between the two groups.

**Conclusion:**

Our findings indicate that cTBS of the right pSTG may improve language production by suppressing intrinsic activity of the right fronto-thalamic-cerebellar circuit and enhancing the involvement of the right temporoparietal region.

## Introduction

Aphasia, one of the most common impairments following stroke, is usually caused by lesions affecting the network of cortical and subcortical structures. Poststroke aphasia (PSA), characterized by deficits in spontaneous speech, auditory comprehension, repetition, naming, etc., is associated with prolonged hospitalization, decreased quality of life, and increased mortality (Lazar and Boehme, 2017). Thus, the treatment of PSA needs to be further explored.

In addition to traditional speech and language therapy (SLT), repetitive transcranial magnetic stimulation (rTMS), one of the most promising noninvasive brain stimulation (NIBS) modalities, has emerged as an ideal method to improve the language function of patients with PSA (Ren et al., 2014; Kapoor, 2017; Gholami et al., 2022). rTMS can modulate the excitability and activity of targeted cortical regions, thereby promoting the reorganization of functional networks (Kapoor, 2017; Yoo et al., 2020; Jargow et al., 2021). Continuous theta burst stimulation (cTBS) is a novel rTMS method that reduces excitability. It has shorter stimulation times and better therapeutic effects than conventional paradigms (Bai et al., 2022; Chen et al., 2022). A recent study showed that cTBS over the right pars triangularis of individuals with chronic PSA enhanced word retrieval by facilitating phonological access (Harvey et al., 2019). Yoo et al. (2020) delivered neuronavigated cTBS over the left pars opercularis in healthy subjects, resulting in significantly increased phase synchronization of TMS-evoked potentials (TEPs) in language areas between the two hemispheres and within the left hemisphere. These results may have implications for the development of a cTBS protocol to assist language rehabilitation in patients with PSA.

Given interhemispheric competition, inhibiting the healthy hemisphere will promote the recovery of the lesioned hemisphere. Inhibitory low-frequency rTMS targeting the pars triangularis, an area in the right posterior inferior frontal gyrus (pIFG), has positive effects in treating aphasia (Ren et al., 2014; Kapoor, 2017; Hu et al., 2018). The right posterior superior temporal gyrus (pSTG), homotopic to Wernicke’s area in the left hemisphere, is a critical area for semantic processing and is thus another optimal target for rTMS stimulation. However, few studies have targeted the pSTG in aphasic patients. A randomized sham-controlled study found significant improvement in auditory comprehension and repetition in patients with subacute global aphasia following 3 weeks of low-frequency rTMS over the right pSTG coupled with SLT (Ren et al., 2019). Kawamura et al. administered low-frequency rTMS over the right pSTG to a patient with severe aphasia due to injury of the left IFG and demonstrated subsequent improvement in language perception (Kawamura et al., 2019). Moreover, significant activations of the left and right temporal gyrus, left posterior cingulate gyrus, and left thalamus during a language task following rTMS over the pSTG were observed by functional magnetic resonance imaging (fMRI; Kawamura et al., 2019). A pilot study by Versace demonstrated that excitatory intermittent TBS (iTBS) over Wernicke’s area led to transient facilitation of auditory comprehension in patients with chronic fluent aphasia (Versace et al., 2019). However, these studies have several limitations, including the lack of exploration of underlying mechanisms and insufficient sample size.

However, research is lacking on whether cTBS of the contralesional pSTG improves language outcomes in patients with PSA, and the underlying mechanisms are still unknown. Resting-state fMRI (rs-fMRI) has proven to be a feasible tool for the exploration of networkwide lesion effects with the potential to provide insight into network reconfiguration following stroke and is increasingly being utilized to investigate PSA (Ilkhani et al., 2018; Allendorfer et al., 2021a,b). Therefore, the present study was conducted to determine whether inhibitory cTBS over the right pSTG (coupled with SLT) benefits patients with subacute aphasia and to explore the remodeling mechanism of neural plasticity. Specifically, the current study aimed to explore changes in intrinsic neural activity by examining the outcomes of rs-fMRI, including whole-brain analysis of fractional amplitude of low-frequency fluctuations (fALFF) and functional connectivity (FC).

## Materials and methods

### Participants

**Figure 1:**
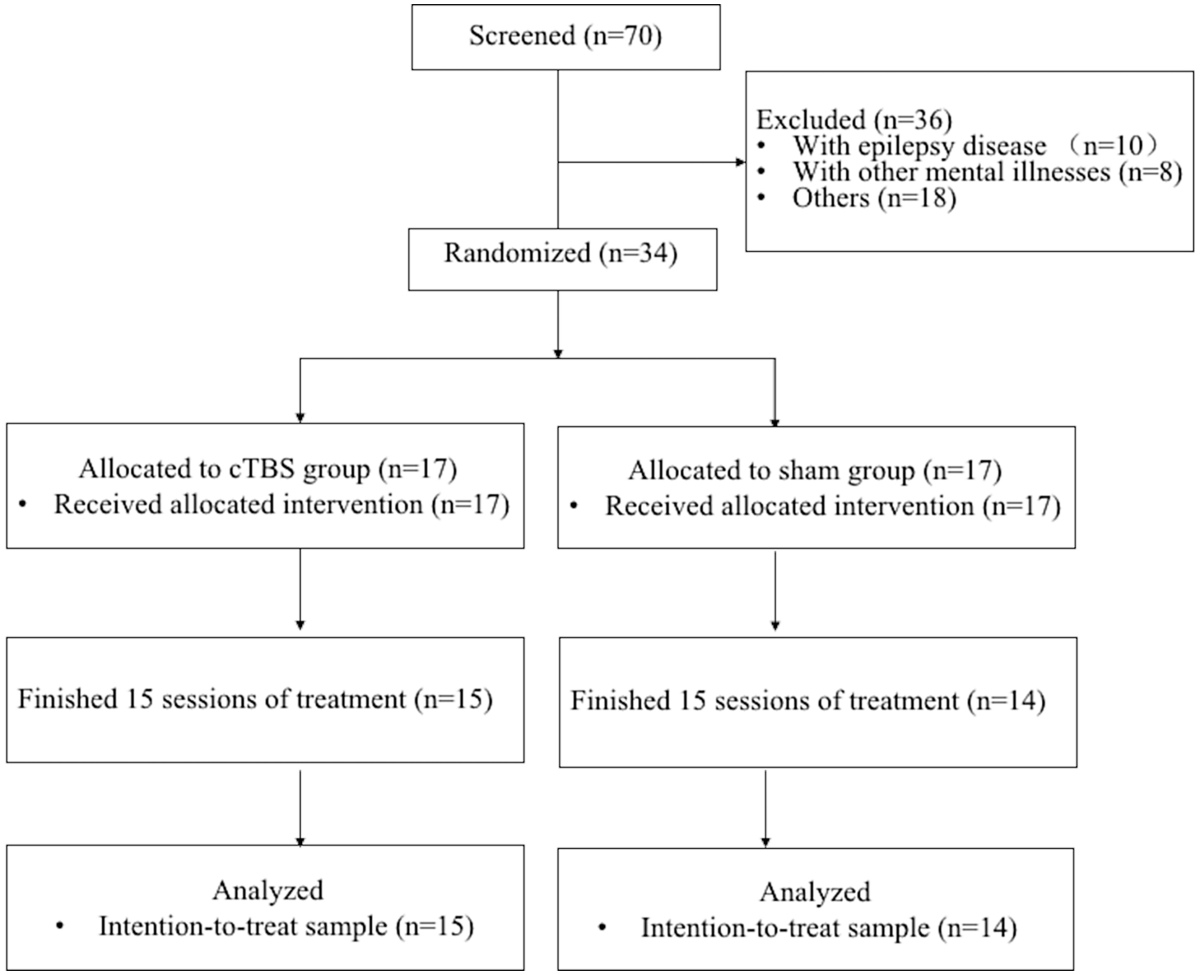
CONSORT diagram of patient flow through the study.

A total of 34 participants with aphasia were recruited from Wuxi Mental Health Center (Wuxi Central Rehabilitation Hospital). Details about the study design and data collection are shown in Figure 1. The inclusion criteria were as follows: (1) provided written informed consent; (2) aged 45 to 75 years; (3) were right-handed (as assessed by the Edinburgh Handedness Inventory; Oldfield, 1971), native Chinese speakers without professional vocal or instrumental training; (4) experienced their first middle cerebral artery (MCA) stroke on the left side, with an MRI-identified lesion site; (5) were 4–12 weeks poststroke; (6) had aphasia confirmed by the Chinese version of Western Aphasia Battery (WAB) scores; and (7) had at least elementary education and normal or corrected-to-normal vision and hearing. The exclusion criteria were as follows: (1) history of epilepsy, drug abuse, or neuropsychiatric diseases; (2) speech impairment or severe dysarthria before the onset of this disease; (3) contraindications to TMS or fMRI (e.g., skull defects or skin lesions at the site of stimulation, intracranial implants, pacemakers, or implanted drug pumps); (4) other neurological diseases; and (5) history of neurosurgical treatment.

The present randomized study was approved by the Ethics Committee of Wuxi Mental Health Center (Wuxi Central Rehabilitation Hospital) (No. WXMHCIR2021LLky054) and registered in the Chinese Clinical Trial Registry (No. ChiCTR210052962).

### Procedure

A prospective, randomized, and sham-controlled study was performed. The random allocation sequence was generated by a completely randomized digital table. A blinded investigator randomly assigned patients to the cTBS group and the sham group using numbers in opaque envelopes. The patients in both groups (the cTBS group: *n* = 15; the sham group: *n* = 14) underwent 15 sessions (5 times a week, from Monday to Friday) of real or sham cTBS coupled with 30 min of SLT over 3 weeks. The rehabilitation programs focused on specific training that stimulated various aspects of the language system, such as semantic, syntactic, phonological and motor speech performance.

Language assessment was obtained before and after 3 weeks of real or sham cTBS treatment with the WAB, which consists of the AQ score and subscales of performance on spontaneous speech, auditory verbal comprehension, repetition, and naming. The AQ score reflects the severity of aphasia and can be used to assess the improvement in or worsening of aphasia symptoms. The highest AQ score is 100; the normal range is 98.4–99.6. An AQ score < 93.8 indicates aphasia.

### Image acquisition

MRI scanning was performed with a 3.0 T MAGNETOM Skyra scanner (Siemens, Germany) the day before and after the intervention. A total of 240 brain volumes were obtained using an echo-planar imaging (EPI) pulse fMRI sequence with the following parameters: repetition time (TR) = 2,000 ms; echo time (TE) = 30 ms; 35 slices, field of view (FOV) = 224 mm × 224 mm; slice thickness = 3.5 mm; layer spacing = 0.7 mm; flip angle (FA) = 90°; acquisition matrix = 64 × 64; and voxel size = 3.5 mm × 3.5 mm × 3.5 mm. The parameters of the high-resolution three-dimensional (3D) structural T1-weighted images were as follows: 192 sagittal slices; slice thickness = 1 mm; TR = 6.6 ms; TE = 3.1 ms; and FA = 12°. Each subject was instructed to remain still, close their eyes, and not think of anything in particular before scanning.

### Lesion analysis

The lesions were manually segmented into individual structural images obtained from the T1-weighted sequence using ITK-SNAP tools (v. 3.2) (Yushkevich et al., 2016) and registered to Montreal Neurological Institute (MNI) space using SMP12. The sum of all the individual lesions was used to construct a voxelwise map of the lesions.

### Preprocessing of resting-state functional MRI (rs-fMRI) data

rs-fMRI data were preprocessed on the MATLAB platform (MathWorks, Natick, MA, United States) with DPABI software (v. 6.1) (Yan et al., 2016). The main steps were as follows: (1) conversion of the raw data in DICOM format to NIFTI format using MRIConvert software (v. 2.1.0, Lewis Center, Eugene, OR, United States); (2) removal of the first 10 slices and slice-timing correction of the remaining 230 slices; (3) correction of head motion using Friston 24 parameters; (4) spatial normalization of the functional images to MNI space and resampling to a voxel size of 3 mm × 3 mm × 3 mm; (5) application of a bandpass filter (0.01–0.08 Hz) to each voxel to reduce the influence of low-frequency fluctuations and high-frequency noise (Lv et al., 2018); and (6) smoothing of the functional data with a 6-mm full-width at half-maximum (FWHM) Gaussian kernel. An enantiomorphic approach was performed to replace the lesioned brain tissue with the contralateral mirrored scans during spatial normalization (Nachev et al., 2008).

### Calculation of the fractional amplitude of low-frequency fluctuations (fALFF)

fALFF indicates the amplitude of spontaneous fluctuation in blood oxygen level-dependent (BOLD) signal intensity in a specific brain area in fMRI, reflecting the level of spontaneous activity of a voxel in the resting state (Zou et al., 2008). The time series data were converted into the frequency spectrum using a fast Fourier transform (FFT), and the power spectrum was obtained. In each voxel, the square root of the signal over 0.01–0.08 Hz was calculated. The fALFF values of each voxel were normalized through transformation to z scores.

### Seed-based functional connectivity analyses

Seed-based FC analyses were used to investigate functional changes in the right pSTG and right pars triangularis compared to all the other voxels in the brain. Two 10-mm spherical regions of interest (ROIs) were created based on the Harvard-Oxford Cortical Atlas in the DPABI toolbox (Whitfield-Gabrieli and Nieto-Castanon, 2012; Yan et al., 2016). The MNI coordinates of the right pSTG and right pars triangularis were *x* = 61, *y* = −24, *z* = 1 and *x* = 48, *y* = 30, *z* = 6, respectively (Amad et al., 2017). The time courses of all voxels within a 5-mm sphere around the center coordinate were averaged. Connectivity maps were calculated by calculating the Fisher z-transformed correlation coefficient between the average time course and the time course of each voxel in the whole-brain mask for each subject.

### cTBS protocol

T1-weighted structural imaging data were acquired before cTBS intervention for precise targeting of regions in individuals. The individual stimulation sites around the right pSTG (MNI coordinates *x* = 61, *y* = −24, *z* = 1) were identified in each patient. Individual structural images were loaded into the visor2^™^ TMS neuronavigation system (ANT Neuro, Netherlands) to enable fMRI-guided cTBS intervention. The cTBS intervention was conducted with a Magneiro^®^ (Vishee Medical Technology Co., Ltd., Nanjing, China) with a 70-mm figure-8-shaped stimulation coil. Before intervention, a single-pulse TMS was applied over the right primary motor cortex to determine the active motor threshold (AMT). The AMT was defined as the lowest stimulus intensity that elicited a motor evoked potential (MEP) >200 μV in at least 5–10 consecutive trials, with a 10% maximal voluntary contraction of the first interosseous muscle of the left hand (Koch et al., 2020). Each subject received real or sham cTBS over the right pSTG at 80% of the AMT (Nee and D’Esposito, 2017). The standard cTBS protocol consisted of a burst of three 50-Hz pulses, presented repeatedly at a frequency of 5 Hz, resulting in a total of 600 pulses applied over 40 s. The coil was placed tangentially to the skull with the handle pointing backward and laterally angled at 45° from the sagittal axis during real stimulation. In contrast, the stimulation coil was rotated 90° to place one side of the coil on the target during sham stimulation.

### Statistical analysis

Statistical analysis was performed using SPSS for Windows, version 24.0 (SPSS, Inc., Chicago, IL, United States). The sample size estimation was determined according to a previous randomized controlled study (Thiel et al., 2013). One-way analyses of variance (ANOVAs) were used for continuous data, and the *χ*^2^ test was used for categorical data in group comparisons of baseline demographic characteristics. Between-group differences in WAB score changes were assessed using independent-sample *t* tests and a 2-factor repeated-measures ANOVA.

The overall pre-and posttreatment maps of fALFF and FC values were performed using a 2-factor mixed-effects ANOVA, with time (pre-vs. post-treatment) as a within-subjects factor and treatment (cTBS vs. sham) as a between-subjects factor. Framewise displacement (FD) was included as a covariate. Multiple comparisons across voxels were corrected by a Gaussian random field (GRF) (voxelwise *p* < 0.001, clusterwise *p* < 0.05, two-tailed). Linear regression analysis was used to determine the relationship between changes in WAB scores and fALFF values.

## Results

### Participant characteristics

Thirty-four eligible subjects participated in the study after application of the inclusion and exclusion criteria. Four patients (two from the sham group and two from the cTBS group) withdrew from the trial after providing informed consent and after being allocated to the groups. One patient (from the cTBS group) dropped out because of complications. Thus, a total of 29 participants completed the study (cTBS group: *n* = 15; sham group: *n* = 14) (Figure 1).

The baseline demographic characteristics of all 29 subjects were shown in Table 1. No significant differences between the two groups were observed at baseline in terms of age (*p* = 0.47), sex (*p* = 0.76), time since stroke (*p* = 0.59), or WAB scores (*p* > 0.05).

**Table 1:** Demographic and clinical characteristics of the participants.

	cTBS group	Sham group	Test statistic	*p*
Total n	15	14		
Sex, M/F	13/2	11/3	*χ*^2^ = 0.528	0.768
Mean age, years	65.13 (11.26)	66.33 (13.77)	*t* = −0.745	0.475
Time since stroke, days	42.80 (12.91)	55.50 (21.93)	*t* = 0.547	0.592
Aphasia type, n (%)				
Broca’s	5 (33%)	3 (21%)		
Wernicke’s	4 (27%)	3 (21%)		
Global	6 (40%)	8 (58%)		
Lesion location, n (%)				
Anterior MCA	3 (20%)	2 (14%)		
Posterior MCA	4 (27%)	5 (36%)		
Anterior and posterior MCA	6 (40%)	6 (43%)		
Subcortical	2 (13%)	1 (7%)		
Initial aphasia scores				
WAB-AQ	22.99 (13.24)	17.38 (11.14)	*t* = 1.265	0.217
Spontaneous speech	3.67 (2.35)	2.76 (2.49)	*t* = 1.012	0.320
Comprehension	3.50 (2.09)	2.66 (1.98)	*t* = 1.103	0.280
Naming	1.68 (1.85)	0.98 (0.43)	*t* = 1.379	0.179
Repetition	2.72 (2.05)	2.31 (2.18)	*t* = 0.516	0.610
Time between assessments, days	17 (0.85)	16.71 (0.99)	*t* = 0.836	0.411

Values are presented as the mean and standard deviation (SD; in parentheses) unless otherwise specified. The *p* value refers to that of an independent-samples *t* test.

### Language measures

**Figure 2:**
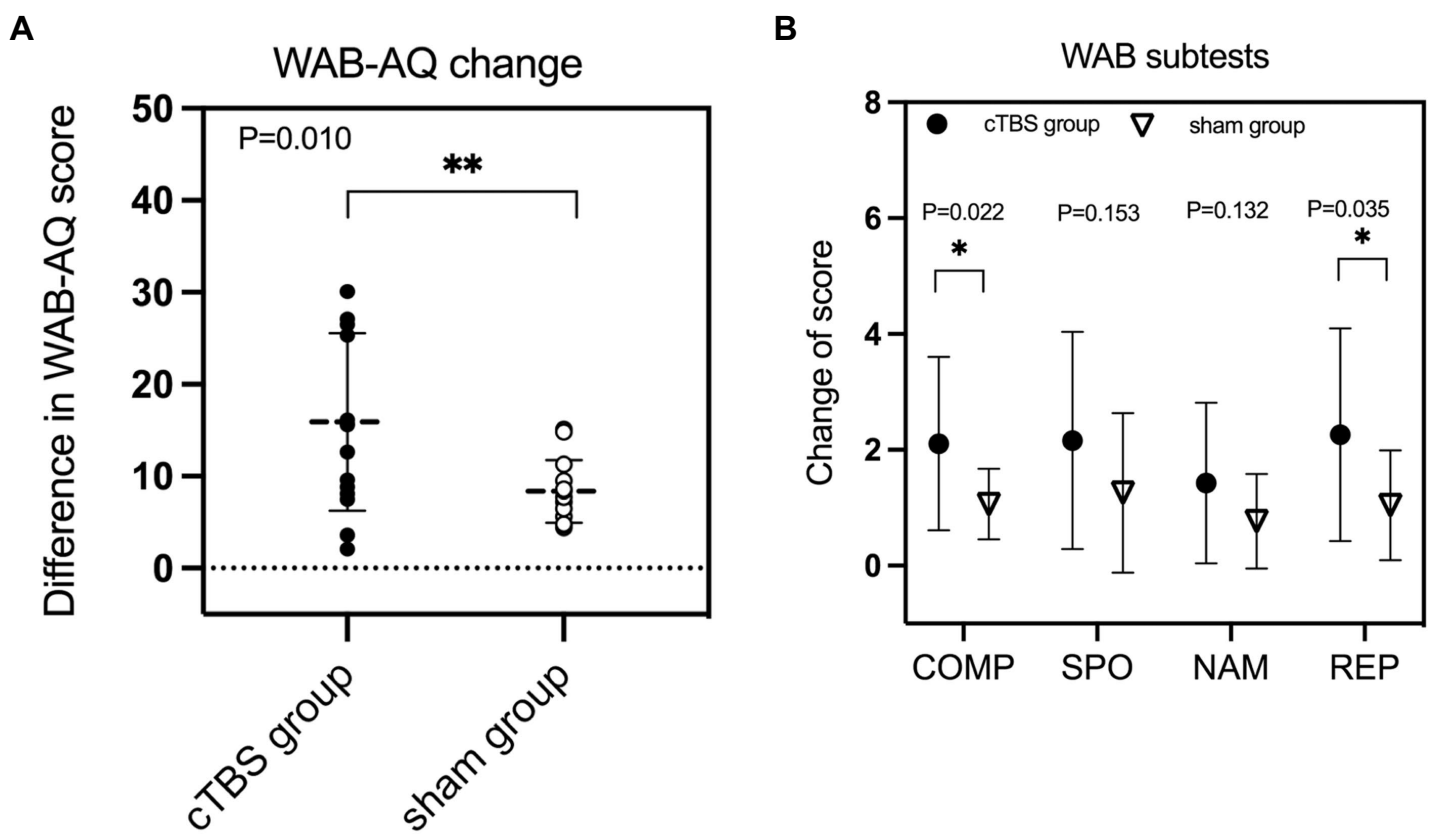
The change in the aphasia quotient (AQ) score of the Western Aphasia Battery (WAB) was significantly greater in the continuous theta burst stimulation (cTBS) group than in the sham group **(A)**. cTBS significantly improved comprehension (COMP) and repetition (REP) performance but not spontaneous speech (SPO) or naming (NAM) performance **(B)**. *Significant at *p* < 0.05. ** Significant at *p* ≤ 0.01.

A repeated-measures ANOVA revealed a significant group × time interaction effect on WAB-AQ scores [*F*(2,27) = 7.687, *p* = 0.010, *η*^2^ = 0.222] (Table 2). There was a significant main effect of time [*F*(2,27) = 79.172, *p* < 0.001, *η*^2^ = 0.746], but no significant main effect of treatment [*F*(2,27) = 3.559, *p* = 0.070, *η*^2^ = 0.116]. Independent-samples t tests showed that the change in WAB-AQ scores was significantly higher in the cTBS group than in the sham group (*t* = 2.773, 95% CI = 1.966 to 12.162, *p* = 0.010) (Figure 2).

**Table 2:** Statistical data of WAB score changes with pre-and post-treatment between the cTBS group and the sham group.

Test and variables	Group difference	95% confidence interval	*p* value	*η* ^2^
Repeated-measures ANOVA: WAB scores				
Treatment × time interaction				
WAB-AQ	7.687		0.010*	0.222
Spontaneous speech	2.159		0.153	0.074
Comprehension	5.870		0.022*	0.179
Naming	2.412		0.132	0.082
Repetition	4.925		0.035*	0.154

Column 2 presents the differences between group means on the ANOVA. WAB, Western Aphasia Battery; AQ, aphasia quotient; cTBS, continuous theta burst stimulation; treatment, cTBS vs. Sham; time, pre-vs. post-treatment. *Significant at *p* < 0.05.

Regarding scores on the WAB subscales, there was a significant group × time interaction effect on auditory comprehension [*F*(2,27) = 5.870, *p* = 0.022, *η*^2^ = 0.179] and repetition scores [*F*(2,27) = 4.927, *p* = 0.035, *η*^2^ = 0.154] (Table 2). There was no significant interaction effect on spontaneous speech or naming scores (*p* > 0.10). The changes in auditory comprehension scores (*t* = 2.423, 95% CI = 0.159 to 1.926, *p* = 0.022) and repetition scores (*t* = 2.220, 95% CI = 0.092 to 2.345, *p* = 0.035) were significantly higher in the cTBS group than in the sham group (Figure 2).

### fALFF changes after cTBS

**Figure 3:**
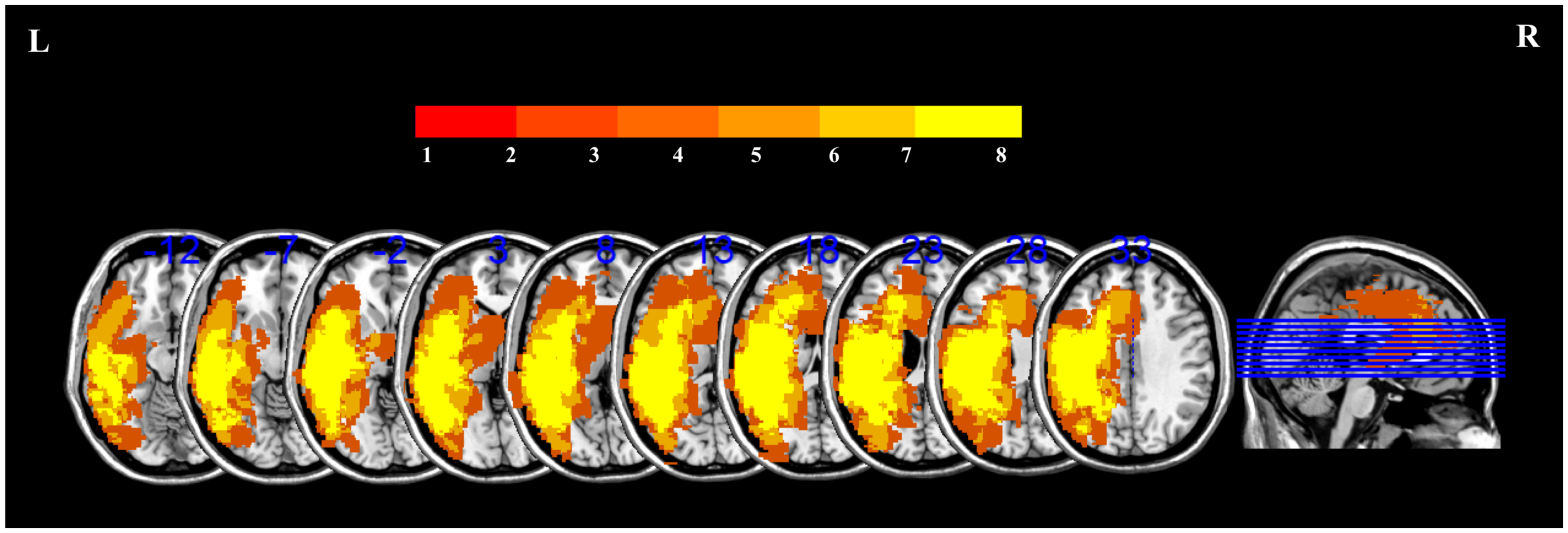
Lesion overlap map for 29 patients with PSA to illustrate the distribution of lesions. The color scale in the spectrograms represents the frequency of injury in a given location.

Figure 3 presents the lesion overlap map and the distribution of lesions for all patients.

**Figure 4:**
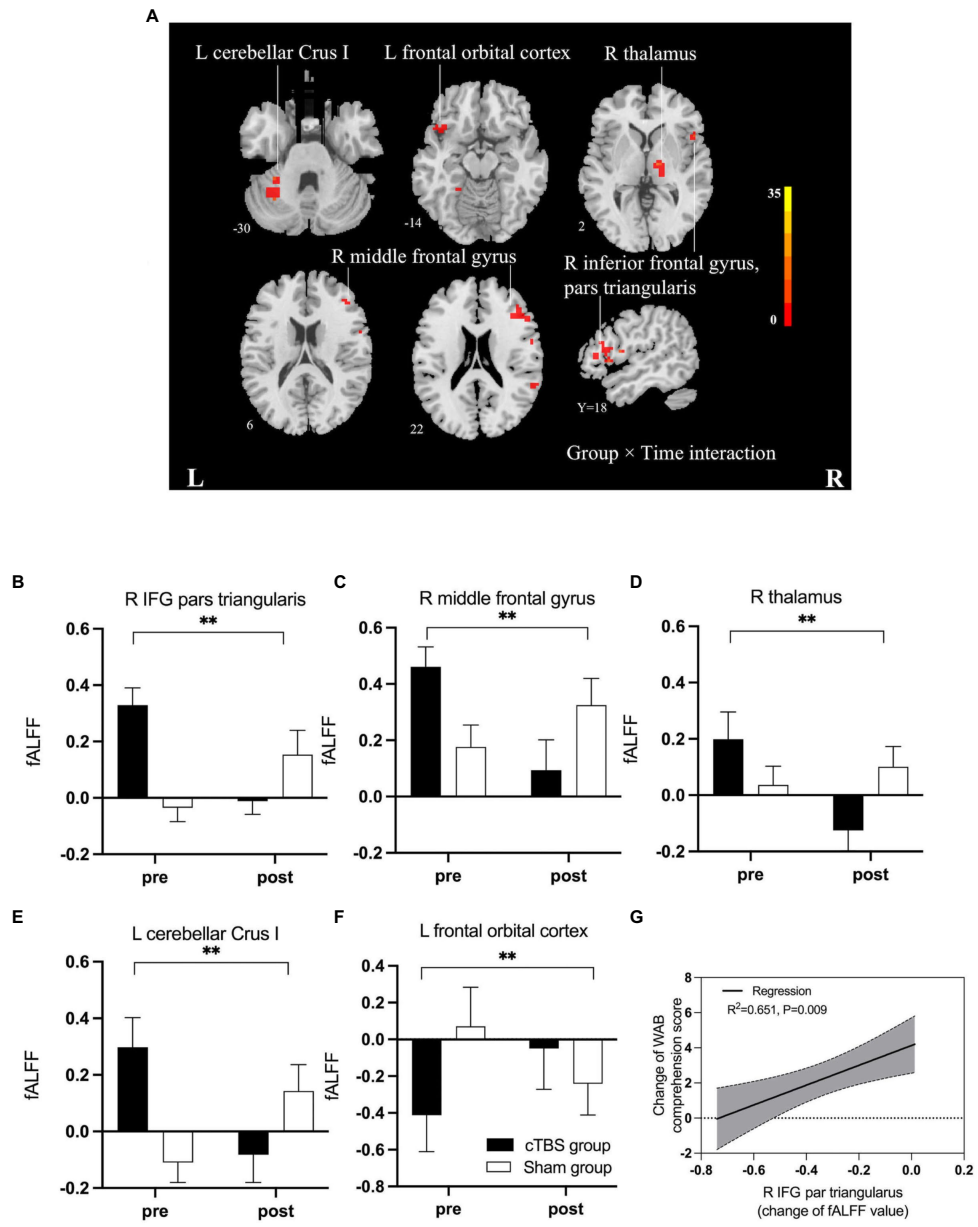
**(A)** F map of differences in spontaneous activity pre-and post-treatment between the cTBS group and sham group. Brilliant colors (red to bright yellow) indicate a greater significant difference over pre-and post-treatment between the cTBS and sham groups (group × time interaction). **(B–F)** Fractional amplitude of low-frequency fluctuations (fALFF) decreased significantly after cTBS in the right pars triangularis, left cerebellar crus I, right middle frontal gyrus, and right thalamus and increased significantly after cTBS in the left orbitofrontal cortex. We used a 2-factor mixed-effects ANOVA. ** indicates *p* < 0.001 in **(B–F)**. Bars show means, and error bars represent the standard error of the mean (SEM). **(G)** The regression analysis showed a significant linear correlation between changes in fALFF in the right pars triangularis and changes in the WAB comprehension score. IFG, inferior frontal gyrus; WAB, Western Aphasia Battery.

Figure 4 and Table 3 indicate the clusters with significant between-group differences in fALFF before and after cTBS intervention. A 2-factor mixed-effects ANOVA showed that the great significant difference in group and time interaction occurred in brain regions including the pars triangularis of the inferior frontal gyrus (IFG), right middle frontal gyrus, right thalamus, left orbitofrontal cortex and left cerebellar Crus I (corrected by GRF, voxelwise *p* < 0.001, clusterwise *p* < 0.05, two-tailed). Specifically, the intrinsic activity indexed by the fALFF values in the right pars triangularis of IFG, right middle frontal gyrus, right thalamus, and left cerebellar Crus I decreased significantly from pre-to post-treatment in the cTBS group compared to those in the sham group. In contrast, the fALFF value of the left orbitofrontal cortex increased significantly from pre-to post-treatment in the cTBS group compared to the sham group.

**Table 3:** Brain regions with significantly different values of fALFF in the intragroup (pre-and post-treatment) and intergroup (cTBS and sham groups) comparisons with group × time interactions (GRF, voxelwise *p* < 0.001, clusterwise *p* < 0.05, two-tailed).

Brain regions	BA	Voxels	MNI	*F* values
Inferior frontal gyrus, pars triangularis (R)	BA45	113	48, 30, 6	9.795
Cerebellar Crus I (L)	/	106	−43, −60, −36	7.214
Middle frontal gyrus (R)	BA48	115	42, 27, 24	11.466
Orbitofrontal cortex (L)	BA38	96	−42, 21, −12	12.213
Thalamus (R)		97	12, −12, 3	18.755

A linear regression analysis was performed to test whether intrinsic regional activity, represented by changes in fALFF, correlated with clinical recovery. The regression analysis revealed that the change in WAB comprehension scores were significantly associated with the change in the fALFF value of the right IFG pars triangularis in both groups (*R*^2^ = 0.651, *p* = 0.009).

### Functional connectivity

**Figure 5:**
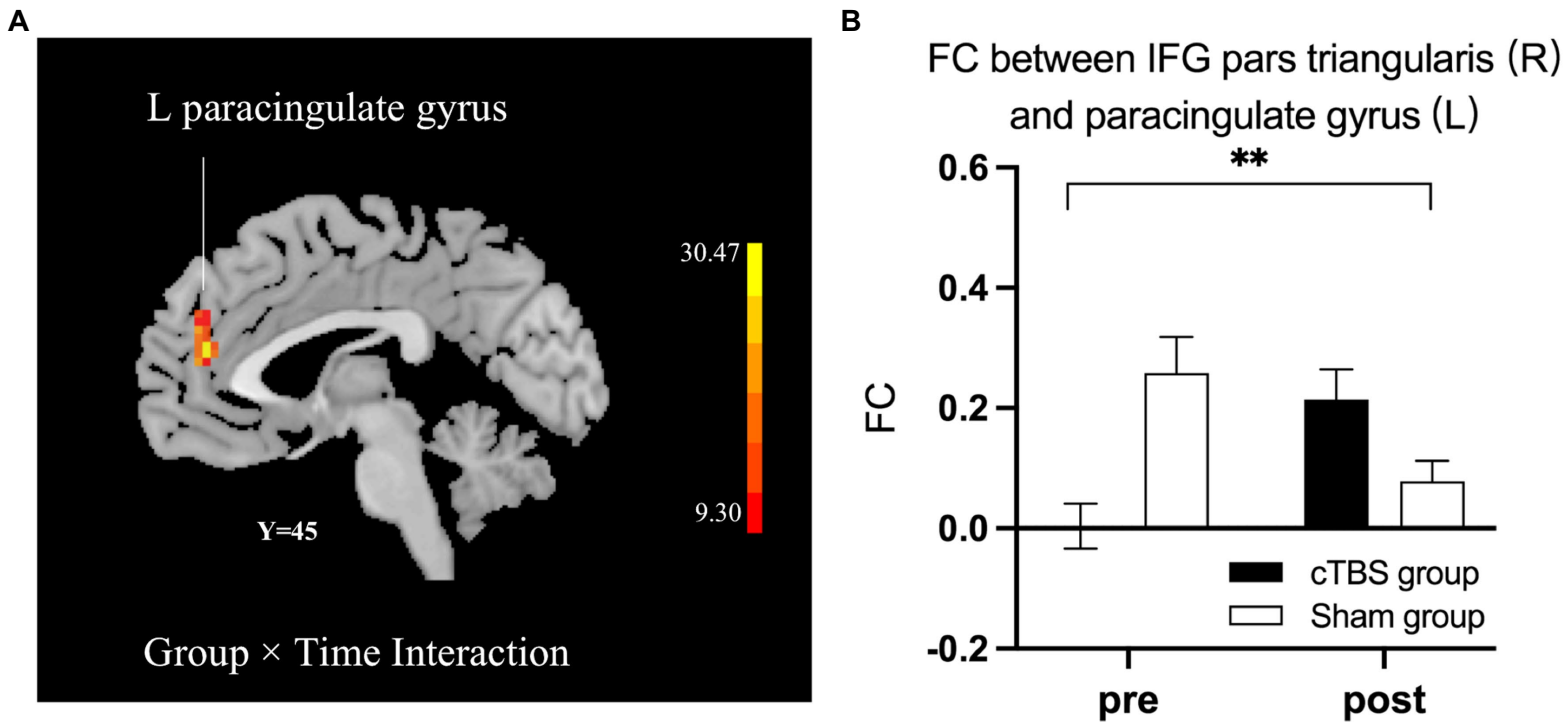
cTBS-induced changes in intrinsic functional connectivity (FC) between the right pars triangularis and the left paracingulate gyrus **(A)**. The cTBS group demonstrated increased FC between the right pars triangularis and left paracingulate gyrus after treatment, but FC between these regions was reduced after treatment in the sham group. Bars show means, and error bars represent the standard error of the mean (SEM). We used a 2-factor mixed-effects ANOVA. ** indicates *p* < 0.001 in **(B)**.

**Figure 6:**
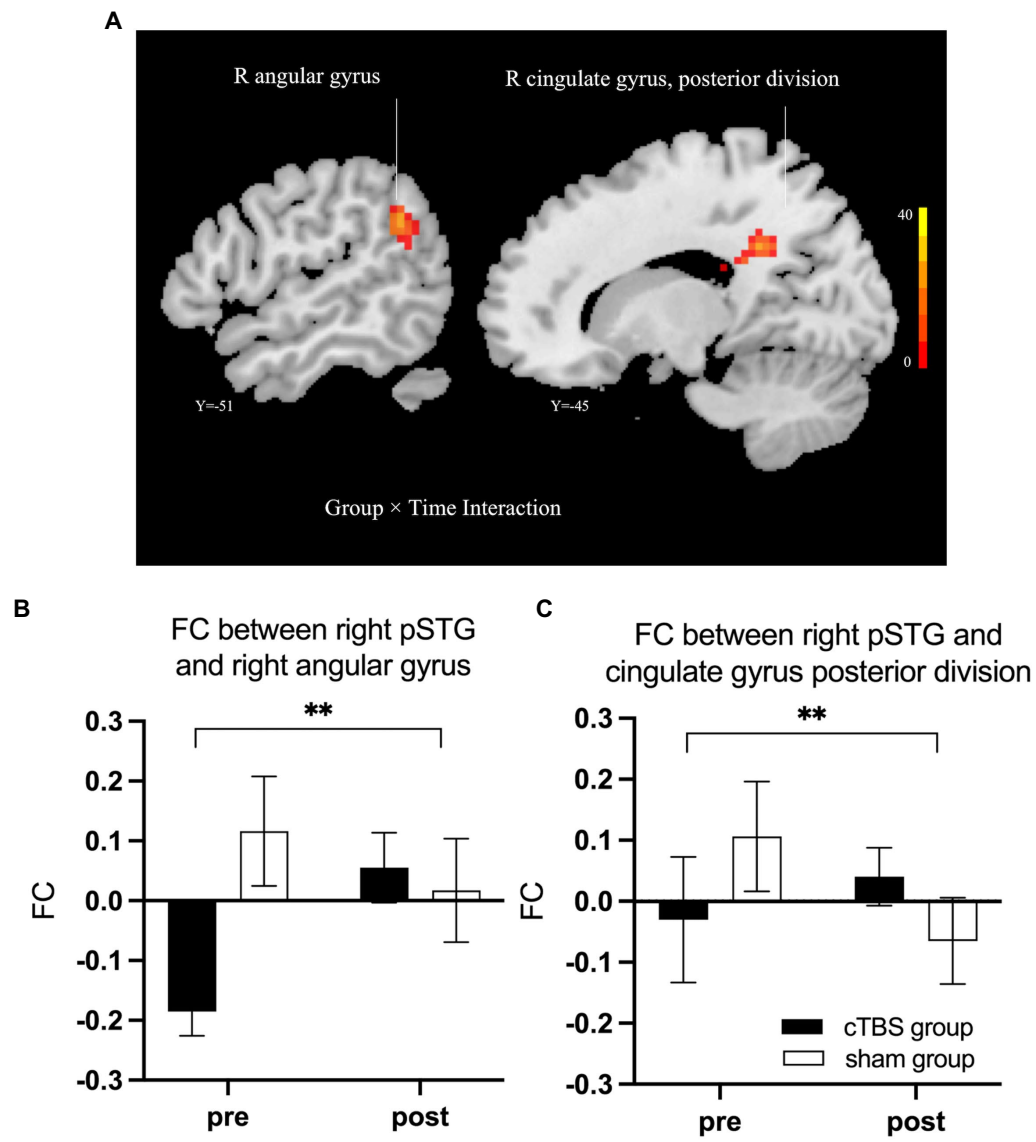
cTBS-induced changes in intrinsic functional connectivity (FC) of the right posterior superior temporal gyrus (pSTG) with the right angular gyrus and the posterior cingulate gyrus **(A)**. The cTBS group demonstrated increased FC of the right pSTG with the right angular gyrus and posterior cingulate gyrus after treatment; however, in the sham group, the FC between these areas was reduced after treatment. Bars show means, and error bars represent the standard error of the mean (SEM). We used a 2-factor mixed-effects ANOVA. ** indicates *p* < 0.001 in **(B,C)**.

We defined the right IFG pars triangularis as the seed region based on the results of the linear regression analysis. A 2-factor mixed-effects ANOVA showed that the cTBS group demonstrated increased FC between the right IFG pars triangularis and left paracingulate gyrus but reduced FC of these regions in the sham group (Figure 5). The right pSTG, the stimulation site, was set as the other seed region. The FC of the right pSTG with the right angular gyrus and posterior cingulate gyrus was significantly increased after treatment in the cTBS group but was reduced after treatment in the sham group (Figure 6).

## Discussion

Our prior work documented the effectiveness of low-frequency rTMS (LF-rTMS) over the right pSTG for improving language performance in stroke patients with aphasia (Ren et al., 2019). The present study revealed that cTBS over the right pSTG (homotopic to Wernicke’s area) coupled with SLT improved language performance, especially auditory comprehension and repetition, in patients with PSA, as demonstrated by the increase in WAB scores on the AQ, auditory comprehension and repetition. These findings expand potential stimulation targets for effective intervention beyond anterior language areas, in which most studies targeted areas homotopic to Broca’s area, to posterior temporal language areas in the right hemisphere. Notably, the observed improvements in our study were paralleled by suppression of spontaneous neural activity in the right fronto-thalamic-cerebellar circuit and an increase in FC of the right temporoparietal lobe, as measured by rs-fMRI. Therefore, this study may provide a fruitful target for improving the language function of auditory comprehension as well as valuable insight into how cTBS reorganizes language networks in aphasic patients.

We found that the spontaneous neural activity of the right pars triangularis was significantly suppressed after patients received inhibitory cTBS over the right pSTG; this decrease was closely related to the improvement in auditory comprehension. Therefore, cTBS of the right pSTG may downregulate the activity of the right pars triangularis through structural or functional connections between the two major nodes, such as the arcuate fasciculus. The right pars triangularis, the homotopic area to Broca’s area frequently used as a stimulation target, was inhibited in most of the previous studies addressing interhemispheric imbalance by decreasing activity in the right hemisphere or increasing activity in the left hemisphere (Thiel et al., 2013; Heikkinen et al., 2018; Lee et al., 2022; Zumbansen et al., 2022). Our fALFF results provided compelling evidence for cTBS-induced reduction in spontaneous neural activity not only in the right frontal language regions but also in subcortical regions, including the right thalamus and the left cerebellum, in aphasic patients compared with those who received sham treatment. This result is consistent with that of a positron emission tomography (PET) study, which showed hypometabolism in the right hemisphere and left cerebellum after stimulating Wernicke’s area in a patient with crossed aphasia in dextral areas (Lu et al., 2014). Additionally, the previous clinical trial illustrated that clusters in the right thalamus exhibited suppressed fALFF in patients who received inhibitory LF-rTMS of the right pars triangularis (Lee et al., 2022), which was in accordance with our findings.

We noticed that a similar synchronized decline in fALFF was present in the frontal and subcortical areas belonging to the contralesional cerebro-cerebellar circuit, including the right IFG pars triangularis, right middle frontal gyrus, right thalamus and left cerebellar hemisphere. This finding demonstrates that the inhibitory stimulation of cTBS over the right pSTG downregulated the intrinsic neural activity in the right frontal-thalamic-cerebellar circuit compared with that of the sham group, which might be due to the major neuroplastic alteration of the contralesional cerebro-cerebellar system not only in response to stroke and left cerebral hemispheric glioma involving the language network (Wessel and Hummel, 2018; Zhang et al., 2018) but also following the inhibitory stimulation of cTBS over the contralesional hemisphere cortex. The cerebro-cerebellar circuit, which transports information between the contralateral cerebellum and the ipsilateral cerebral cortex *via* the thalamus, is properly organized in a crossing pattern (Habas et al., 2009; Stoodley and Schmahmann, 2010; Buckner, 2013; De Smet et al., 2013). A number of anatomical, clinical, and functional imaging studies have confirmed that the left cerebro-thalamic-cerebellar circuit is involved in language processing with strong lateralization (Marien et al., 2001; Baillieux et al., 2010; Stoodley and Schmahmann, 2010; Mariën and Beaton, 2014; Schwartze and Kotz, 2016; Mariën and Borgatti, 2018). Additionally, a previous task-state fMRI study in which aphasic patients exhibited stronger lateralization to the right cerebellar hemisphere during a semantic decision/tone decision task after 3 weeks of iTBS over the ipsilesional hemisphere (Szaflarski et al., 2021). These findings highlight the potentially critical role of the cerebro-cerebellar circuit in the remodeling process following TBS intervention in the cerebral language network. Moreover, the spontaneous neural activity in the left orbitofrontal cortex significantly increased after 3 weeks of cTBS treatment in aphasic patients. The orbitofrontal cortex has long been recognized as being involved in affection and decision-making (Rolls and Grabenhorst, 2008). Its action during language processing remains unknown. However, the orbitofrontal cortex has been found to be coactivated with a network of prefrontal regions and the bilateral thalamus, which are involved in cognitive functions including language and memory (Zald et al., 2014). Accordingly, we can infer that the increased neural activity in the left orbitofrontal cortex may be viewed as the recruitment of intra-hemispheric language-network plasticity following the cTBS intervention.

The static FC of the right pars triangularis with the left paracingulate gyrus and of the right pSTG with the right angular gyrus and posterior cingulate gyrus was increased after treatment in the cTBS group but decreased after the sham intervention. These increased FC areas belong to the domain-general cognitive network, which implies that the domain-general cognitive network may be associated with recovery from aphasia (Brownsett et al., 2014). Cognitive deficits in aphasic patients are difficult to measure because of challenges with verbal communication (Arheix-Parras et al., 2021). The anterior paracingulate gyrus participates in speech processing through its connection to the prefrontal cortex, as confirmed by a previous fMRI study using word generation tasks (Crosson et al., 1999). Moreover, the angular gyrus may be included in the “language association areas,” as shown by the activation of the bilateral angular gyri (BA39) and the other language-related cortices following language tasks in a meta-analysis of fMRI data (Rosselli et al., 2015). The current study revealed that cTBS enhanced the involvement of the right angular cortex and the posterior cingulate gyrus in language processing, as reflected by the increased FC and improved clinical-assessment scores. This result is consistent with previous findings regarding structural and functional connectivity and supports the integrative role of the angular gyrus in language functions (Margulies and Petrides, 2013; Sroka et al., 2015; Ardila et al., 2016; Yourganov et al., 2016). A connectome-based analysis reported that the connectivity of the temporoparietal junction (TPJ), a multimodal region critical for language tasks, could predict repetition scores, as with, for example, the connectivity of the TPJ with the angular gyrus and pSTG (Yourganov et al., 2016).

However, this study also has some noteworthy limitations. We did not conduct subgroup analysis of aphasia subtypes because of the relatively small sample size. In future studies, the aphasia subtype and severity of language impairment should be considered during random assignment and treatment. Additionally, long-term linguistic improvement and neural reorganization could not be observed because outcomes were measured immediately after the intervention. Long-term follow-up research could supplement these findings and provide a more comprehensive understanding of brain remodeling.

## Conclusion

To the best of our knowledge, this is the first randomized, sham-controlled clinical trial to investigate the effectiveness and underlying neural mechanism of cTBS over the right pSTG to treat subacute stroke patients with aphasia. Our data provide preliminary evidence that inhibitory cTBS of the right pSTG combined with SLT boosts the recovery of language performance, especially auditory comprehension and repetition. Moreover, the rs-fMRI data showed that the recovery of language function was associated with inhibition of the right fronto-thalamic-cerebellar circuit and increases in the neuroplasticity of the right temporoparietal lobe. Future studies with large samples are needed to elucidate the precise mechanisms underlying language recovery in the fronto-thalamic-cerebellar circuit. However, our results further optimize the TMS protocol and clarify the role of neural remodeling in the right hemisphere during aphasia recovery.

## Data availability statement

The raw data supporting the conclusions of this article will be made available by the authors, without undue reservation.

## Ethics statement

The studies involving human participants were reviewed and approved by the Ethics Committee of Wuxi Mental Health Center (Wuxi Central Rehabilitation Hospital; No. WXMHCIR2021LLky054). The patients/participants provided their written informed consent to participate in this study.

## Author contributions

CR and GZ designed the study. CR, KZ, XX, and YJ drafted the protocol. KZ coordinated the trial. HF, FG, and LB were responsible for data acquisition and clinical evaluation. GH and BS administered the interventions. CR, KZ, and GZ revised the manuscript. All authors read and approved the final manuscript.

## Funding

This work was supported by the Natural Science Foundation of Jiangsu Province (no. BK20201138), the Social Development Project of Jiangsu Province (no. BE2022700), the Wuxi Taihu Talent Project (no. WXTTP2020008), and the Top Talent Support Program for Young and Middle-Aged People of Wuxi Health Committee and General Project from Wuxi Health Commission (no. MS201911).

## Conflict of interest

The authors declare that the research was conducted in the absence of any commercial or financial relationships that could be construed as a potential conflict of interest.

## Publisher’s note

All claims expressed in this article are solely those of the authors and do not necessarily represent those of their affiliated organizations, or those of the publisher, the editors and the reviewers. Any product that may be evaluated in this article, or claim that may be made by its manufacturer, is not guaranteed or endorsed by the publisher.
